# Multiple Benefits of Plasmid-Mediated Quinolone Resistance Determinants in *Klebsiella pneumoniae* ST11 High-Risk Clone and Recently Emerging ST307 Clone

**DOI:** 10.3389/fmicb.2019.00157

**Published:** 2019-02-12

**Authors:** Judit Domokos, Ivelina Damjanova, Katalin Kristof, Balazs Ligeti, Bela Kocsis, Dora Szabo

**Affiliations:** ^1^Institute of Medical Microbiology, Semmelweis University, Budapest, Hungary; ^2^National Public Health Institute, Budapest, Hungary; ^3^Institute of Laboratory Medicine, Clinical Microbiology Laboratory, Semmelweis University, Budapest, Hungary; ^4^Faculty of Information Technology and Bionics, Pázmány Péter Catholic University, Budapest, Hungary

**Keywords:** international clones, multi-drug resistance, whole genome sequence analysis, gene expression, plasmid-mediated quinolone resistance

## Abstract

International high-risk clones of *Klebsiella pneumoniae* are among the most common nosocomial pathogens. Increased diversity of plasmid-encoded antimicrobial resistance genes facilitates spread of these clones causing significant therapeutic difficulties. The purpose of our study was to investigate fluoroquinolone resistance in extended-spectrum beta-lactamase (ESBL)-producing strains, including four *K. pneumoniae* and a single *K. oxytoca*, isolated from blood cultures in Hungary. Whole-genome sequencing and molecular typing including multilocus sequence typing (MLST) and pulsed-field gel electrophoresis (PFGE) were performed in selected strains. Gene expression of plasmid-mediated quinolone resistance determinants (PMQR) was investigated by quantitative-PCR. MLST revealed that three *K. pneumoniae* strains belonged to ST11 and one to ST307 whereas *K*. *oxytoca* belonged to ST52. The isolates harbored different β-lactamase genes, however, all *K. pneumoniae* uniformly carried *bla*_CTX-M-15_. The *K. pneumoniae* isolates exhibited resistance to fluoroquinolones and carried various PMQR genes namely, two ST11 strains harbored *qnrB4*, the ST307 strain harbored *qnrB1* and all *K. pneumoniae* harbored *oqxAB* efflux pump. Levofloxacin and moxifloxacin MIC values of *K. pneumoniae* ST11 and ST307 clones correlated with *qnr* and *oqxAB* expression levels. The *qnrA1* carrying *K. oxytoca* ST52 exhibited reduced susceptibility to fluoroquinolones. The maintained expression of *qnr* genes in parallel with chromosomal mutations indicate an additional protective role of Qnr proteins that can support dissemination of high-risk clones. During development of high-level fluoroquinolone resistance, high-risk clones retain fitness thus, enabling them for dissemination in hospital environment. Based on our knowledge this is the first report of ST307 clone in Hungary, that is emerging as a potential high-risk clone worldwide. High-level fluoroquinolone resistance in parallel with upregulated PMQR gene expression are linked to high-risk *K. pneumoniae* clones.

## Introduction

International high-risk clones of *Klebsiella* *pneumoniae* are among the most common Gram- negative pathogens. In addition to community-acquired infections, it has been known for decades that due to their ability to spread rapidly in hospital environment, these bacteria can cause several outbreaks. Multi-drug resistant (MDR) *K. pneumoniae* emerged and dramatically increased prevalence of nosocomial infections while *K. oxytoca* has been isolated in hospital infections with less frequency (Podschun and Ullmann, 1998; Kang et al., 2006; Zhou et al., 2016).

Multi-drug resistant *K. pneumoniae* acquires various resistance mechanisms that confer antibiotic resistance to commonly used antibiotics. Among the most frequent resistance mechanisms are extended-spectrum β-lactamases (ESBLs), plasmid-mediated AmpC enzyme (pAmpCs), carbapenemases, plasmid-mediated quinolone resistance (PMQR) genes, aminoglycoside-modifying enzymes (AMEs), as well as exogenously acquired 16S rRNA methyltransferase that have been detected in clinical isolates (Yan et al., 2002; Ko et al., 2010; Cao et al., 2014; Bi et al., 2017). Presence of PMQR genes including *qnr* determinants, *aac(6*′*)-Ib-cr, qepA* and *oqxAB* efflux pumps confer reduced susceptibility to fluoroquinolones and facilitate selection of fluoroquinolone resistance in Enterobacterales (Rodríguez-Martínez et al., 2011; Carattoli, 2013). High-risk *K. pneumoniae* clones have acquired these antibiotic resistance determinants, that enabled them to increase their pathogenicity and survival skills. These clones have tenacity and flexibility to accumulate resistance determinants and they have contributed to disseminate global multi-drug resistance (Woodford et al., 2011). Consequently, increased diversity of plasmid-encoded antimicrobial resistance genes facilitates spread of these clones, causing significant therapeutic difficulties.

Multi-drug resistant *K. pneumoniae* strains mainly belong to certain sequence types (ST) namely, ST11, ST14, ST15, ST37, ST101, ST147, ST258, ST336, ST340, and ST874. These represent high-risk international clones that played major role in dissemination in hospital settings and increased frequency in nosocomial infections (Damjanova et al., 2008; Hrabák et al., 2009; Baquero et al., 2013; Munoz-Price et al., 2013; Rodrigues et al., 2014; Gonçalves et al., 2017). Among these clones ST258 has been reported as a hybrid clone that was created by a large recombination event between ST11 and ST442 (Mathers et al., 2015).

International high-risk *K. pneumoniae* ST11 has been frequently detected worldwide as a successful pathogen being associated with important co-resistance and virulence factors (Damjanova et al., 2008; Andrade et al., 2014). However, in recent years, new drug-resistant lineages have emerged internationally and among them, KPC-producing *K. pneumoniae* ST307 has been recognized in the United States which was initially associated with production of CTX-M-15 (Castanheira et al., 2013). Later on, this clone has been reported in several countries including Italy, United Kingdom, Columbia, Pakistan, Morocco, Korea, Tunisia, China, Serbia (Habeeb et al., 2013; Girlich et al., 2014; Gona et al., 2014; Park et al., 2015; Ocampo et al., 2016; Mansour et al., 2017; Novović, 2017; Villa et al., 2017; Xie et al., 2017).

Recent studies related to dissemination and antibiotic resistance of *K. pneumoniae* clones clearly showed that “fitness cost advantage” associated with high-level resistance to fluoroquinolones contributed to emergence of international high-risk *K. pneumoniae* clones. In hospital settings where fluoroquinolones are extensively used, international clones are selected out, allowing dominance over other clones (Tóth et al., 2014; Fuzi, 2016; Fuzi et al., 2017). This capacity will provide these clones increased opportunities to spread as well as allow time to acquire antimicrobial drug resistance determinants from other bacteria (Mathers et al., 2015). Whole-genome sequence analysis contributes to detect markers of pathogens, therefore in our study the aim was to investigate high-level fluoroquinolone resistance in *K. pneumoniae* high-risk clone ST11 and currently emerging ST307.

## Materials and Methods

### Bacterial Strains

In our preliminary examination, a total of 54 *Klebsiella* strains (53 *K. pneumoniae* and a single *K. oxytoca*) isolated from bloodstream infections of patients treated at intensive care units of Semmelweis University between 2010 and 2014 were collected. Species identification was done by MALDI-TOF/MS (Bruker Daltonics, Bremen, Germany). Minimum inhibitory concentration determination was performed by microdilution method based on EUCAST recommendation. ^1^ All *Klebsiella* strains were resistant to third-generation cephalosporins and showed reduced susceptibility or resistance to fluoroquinolones. All strains were tested for presence of PMQR genes and all of them were ESBL producers by phenotypic test. In this study, selection of strains was done based on the following criteria: (1) presence of *qnr* gene and non-wild type fluoroquinolone MIC values: Kox37 (isolated in 2010); (2) presence of *qnr* gene and high fluoroquinolone MIC values: Kpn33 (isolated in 2010), Kpn47 (isolated in 2014), Kpn125 (isolated in 2013); (3) multiple PMQR gene carriage together with high fluoroquinolone MIC values: Kpn115 (isolated in 2013) (Domokos et al., 2016).

### Multilocus Sequence Typing (MLST)

**FIGURE 1:**
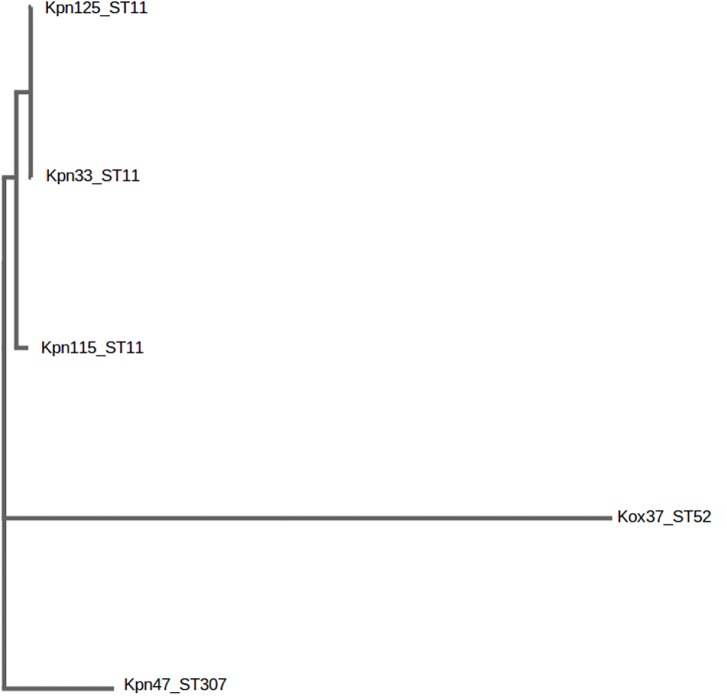
Distance based tree of *K. pneumoniae* ST11, ST307 and *K. oxytoca* ST52 after genome based single nucleotide polymorphism (SNP) analysis. BacWGST, Multiple genome analysis http://bacdb.org/BacWGSTdb/Tools.php.

Genotype of each strain was determined by MLST. The sequences of seven housekeeping genes namely, *gapA, infB, mdh, pgi, phoE, rpoB*, and *tonB* were amplified and directly sequenced. Alleles and sequence types were assigned by using the MLST database^2^ (Diancourt et al., 2005). The distance based relationship between the strains was investigated by BacWGST (Ruan and Feng, 2016) using both the whole-genome MLST and SNP (sequenced based) strategies. Multiple genome analysis was carried out using all the draft genomes of this study and the *HS11286_CP003200_ST11* as a reference genome (Figure 1).

### Pulsed Field Gel Electrophoresis (PFGE) Typing

Clonal relatedness of the four *K. pneumoniae* strains was analyzed by PFGE according to CDC (2000) protocol. Prepared genomic DNA of each strain was digested by *Xba*I restriction endonuclease (Fermentas, ABI, Germany), and DNA fragments were separated in a PFGE CHEF-DR II system (Bio-Rad Laboratories, Hercules, CA, United States). Banding patterns were analyzed by Fingerprinting II Informatix Software (Bio-Rad). *Salmonella enterica* serotype Braenderup H9812 was used as a size marker (Hunter et al., 2005).

### Whole-Genome Sequencing (WGS)

DNA of each strain was extracted by UltraClean Microbial DNA Isolation Kit (Qiagen GmbH, Hilden, Germany). Libraries were prepared using SureSelect QXT Library Prep Kit (Agilent Technologies, Santa Clara, United States). Sequencing was performed on an Illumina MiSeq system using the MiSeq reagent kit v2 generating 250-bp paired-end reads. Trimmomatic (Bolger et al., 2014) was used for preprocessing the WGS data. If the average quality score was below 20 in a sliding window of 4 the adapter sequences and the leading and trailing bases were removed as well as the first 18 bases. Only the reads longer than 50 nucleotides were used for subsequent analysis. *De novo* genome assembly was performed with SPAdes Genome Assembler 3.13.0 (Bankevich et al., 2012). Each assembled genome was accepted for further analysis if it met all of the following quality criteria: (i) average coverage > 30 times, (ii) N50 > 15,000 bases, (iii) maximum contig length > 50,000 bases, and (iv) assembled genome size between 5,000,000 and 6,500,000 bases. Assembled genomes were uploaded to the online bioinformatics tools ResFinder (Zankari et al., 2012), PlasmidFinder (Carattoli et al., 2014) (Center for Genomic Epidemiology, Technical University of Denmark, Lyngby, Denmark) to analyse resistome and plasmid replicon types of the isolates.

### Quantitative PCR (qPCR)

Total RNA of tested strains was isolated by RNeasy Mini Kit (Qiagen) according to the manufacturer’s instructions. The qPCR was carried out in a Step One Real-Time PCR System (Applied BioSystems, Thermo Fisher Scientific). Separate expression of *qnrA1, qnrB1 qnrB4*, *oqxA*, and *oqxB* genes were investigated whereas chromosomal *rpoB* was chosen as housekeeping gene. Set of primers and 6-FAM or VIC labeled probes were designed by Primer Express 3.0 software. All oligonucleotide primers and probes for qPCR are listed in Table 1. Each RNA sample was tested in triplicate. The qPCR was applied in default setting 60°C 30 s; 50°C 5 min; 95°C 10 min; 40 cycles of [95°C 15 s and 60°C 1 min] 60°C 30 s. The C_T_ values of genes of interest were normalized (ΔC_T_) to the C_T_ values of housekeeping gene *rpoB* and the relative expression of each gene of interest was calculated as 2^−ΔC^_T =_ C_T__(geneofinterest)_ – C_T__(rpoB)._

**Table 1:** Primers used for qPCR (F, forward; R, reverse; P, probe).

Gene	Primer sequence
*qnrA1*-F	5′-TTGAGTGACAGCCGTTTTCG-3′
*qnrA1*-R	5′-GCAGCTGACAGTGGCTGAAG-3′
*qnrA1*-P	6-FAM-CTGCCGCTTTTATC-MGB
*qnrB1*-F	5′-GTGCGCTGGGCATTGAA-3′
*qnrB1*-R	5′-CGGAAATCTGCGCCTTGT-3′
*qnrB1-*P	6-FAM-TTCGCCACTGCCGC-MGB
*qnrB4*-F	5′-TGCGCTGGGAATCGAAA-3′
*qnrB4*-R	5′-CGCGAAAATCTGACCCTTGT-3′
*qnrB4*-P	6-FAM-TCGCCACTGCCGGG-MGB
*oqxA*-F	5′-GTCGACGGCTTACAAAAAGTGTT-3′
*oqxA*-R	5′-GCAACGGTTTTGGCGTTAA-3′
*oqxA*-P	6-FAM-ATGCCGGGTATGCC-MGB
*oqxB*-F	5′-CTGGATTTTCCGTCCGTTTAAC-3′
*oqxB*-R	5′-TTGCCTACCAGTCCCTGATAGC-3′
*oqxB*-P	6-FAM-CTGCGCAGCTCGAA-MGB
*rpoB*-F	5′-GTCGCGGCTGAACAAGCT-3′
*rpoB*-R	5′-AACGGCCACTTCGTAGAAGATC-3′
*rpoB*-P	VIC-CTACGGCAGGTAACC-MGB

## Results

In our study, four *K. pneumoniae* and a single *K. oxytoca* were investigated by MLST and PFGE. Three different STs were identified, including ST11 (Kpn33, Kpn115, Kpn125), ST307 (Kpn47), and ST52 (Kox37).

**FIGURE 2:**
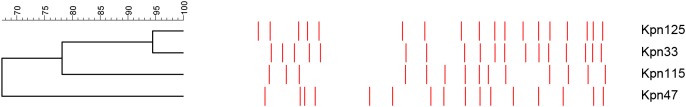
PFGE of *K. pneumoniae* ST11 and ST307.

Pulsed-field gel electrophoresis analysis detected three pulsotypes (PT) among *K. pneumoniae* strains, namely, KP053, S and KP197. Two isolates belonged to KP053 (Kpn33 and Kpn125) and one was detected as S PT (Kpn115). These strains belonged to the ST11 international high-risk clone. By contrast, Kpn47 was classified as KP197 PT (Figure 2).

**FIGURE 3:**
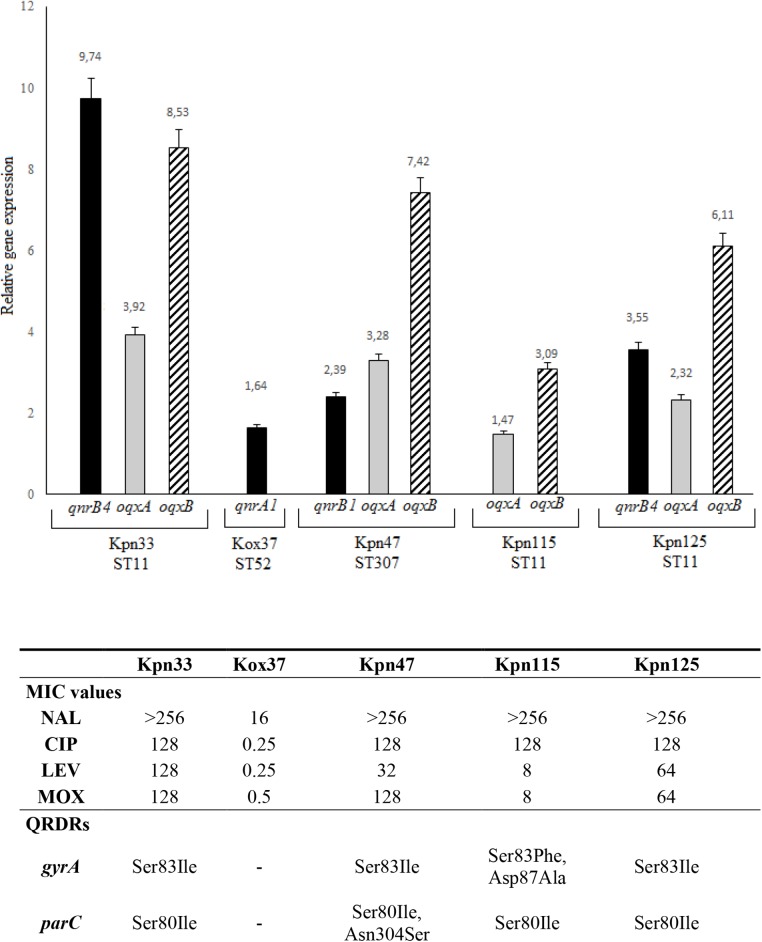
Level of *qnrB4* (Kpn33 and Kpn125), *qnrA1* (Kox37), and *qnrB1, oqxA*, and *oqxB* relative gene expression. QRDRs: quinolone resistance determining regions. All MIC values are in mg/L.

The initial assembled draft genome sequences were 5611026 bp (Kpn33); 6370417 bp (Kox37); 5451744 bp, (Kpn47); 5450412 bp (Kpn115), and 5593358 bp (Kpn125). Seventeen antibiotic resistance genes were found in two ST11 *K. pneumoniae* strains (Kpn33 and Kpn125), twelve were in the third ST11 strain (Kpn115), sixteen resistant genes were in ST307 strain (Kpn47) and ten resistance genes were detected in Kox37. Sequence analysis revealed that the isolates harbored different β-lactamase genes, including *bla*_DHA–1_*, bla*_OXA–1_*, bla*_OXA–2_*, bla*_OXA–9_*, bla*_HV–11_*, bla*_HV–28_, and *bla*_TEM–1A_, *bla*_TEM–1B_*, bla*_OXY–1–3_*, bla*_TLA–1_; and all *K. pneumoniae* strains carried *bla*_CTX–M–15_. Among aminoglycoside resistance genes all isolates were positive for *aac(3)-IIa*. Only Kpn47 carried a tetracycline resistance (*tetA*) gene. Except for Kox37, all strains were identified positive for *fosA* gene nevertheless, *sul1* or *sul2* and trimethoprim resistance (*dfrA12, dfrA14, dfrA29*) genes were detected in four strains. PMQR genes were found in each tested strain namely, in Kpn33 *qnrB4*, in Kox37 *qnrA1*, in Kpn47 *qnrB1*, in Kpn125 *qnrB4.* All *K. pneumoniae* strains harbored *oqxAB* efflux pump and *aac(6*′*)-Ib-cr*, but one of the ST11 strains (Kpn115) carried no *qnr* gene. Presence of phenicol resistance gene (*catA1* or *catB3*) was observed in all strains. Chromosomal mutations conferring fluoroquinolone resistance in *K. pneumoniae* strains were also detected, Ser83Phe and Asp87Ala substitutions were in DNA gyrase subunit A of Kpn115 (ST11), but all other *K. pneumoniae* strains had only Ser83Ile in gyrase while on the other hand all *K. pneumoniae* had a Ser80Ile substitution in DNA topoisomerase IV. Based on the sequencing data, IncFIB, IncFII, and IncR replicons were uniformly present in all ST11 strains. In the case of ST307 IncFIB, IncL/M, IncHI1B were detected. The detected resistance genes and plasmid replicons are listed in Table 2 and Figure 3.

**Table 2:** Distribution of the different resistance genes and plasmid replicons of tested strains.

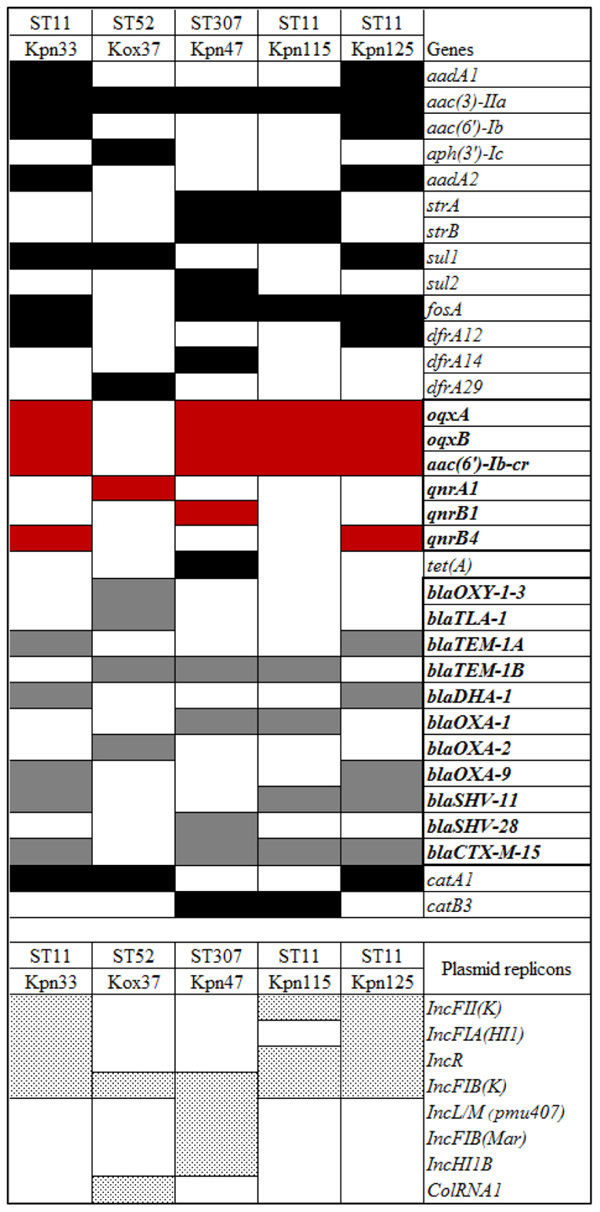

Among *qnr* genes, *qnrB4* of two ST11 strains (Kpn33 and Kpn125) showed 9.74 and 3.55 fold expression, respectively. Interestingly, Kpn33 (ST11) was characterized approximately 3-fold higher expression, compared to the genetically similar Kpn125 (ST11). The lowest expression level (1.64) among *qnr* genes was detected in *K. oxytoca*, that exhibited reduced susceptibility to ciprofloxacin. In the case of *qnrB1* in Kpn47 (ST307), it showed 2.39 fold expression.

Expression of *oqxA* ranged between 1.47 and 3.92 and that of *oqxB* from 3.09 to 8.53. The highest *oqxA* and *oqxB* expressions were observed in Kpn33 (ST11) and Kpn47 (ST307). These were followed by Kpn125 (ST11) and Kpn115 (ST11). Interestingly, Kpn115 a strain of ST11 high-risk clone carried no *qnr* gene moreover, it showed the lowest *oqxAB* expression. It is conspicuous that in every *K. pneumoniae* strain the *oqxB* is expressed 2–3 fold higher than *oqxA*.

## Discussion

International high-risk *K. pneumoniae* ST11 clone has been frequently detected worldwide as a successful pathogen being associated with important virulence (Damjanova et al., 2008; Andrade et al., 2014), and resistance determinants including VIM, NDM and KPC-production (Yan et al., 2002; Kristóf et al., 2010; Qi et al., 2011; Yu et al., 2016; Campana et al., 2017). In our study, all strains of ST11 international high-risk clone carried *bla*_SCTX–M–15_ ESBL that correlates well with earlier studies as the most common global ESBLs are the CTX-M type beta-lactamases in Enterobacterales (Nordmann and Poirel, 2014). Recently, in a Bulgarian study among 82 ESBL-producing *K. pneumoniae* and four *K. oxytoca* CTX-M-15 (87%) was predominant (Markovska et al., 2017). *K. pneumoniae* ST11 has been already reported in Hungary, as a widely disseminated clone in all over the country (Damjanova et al., 2008). In Poland, an inter-regional outbreak was reported that was dominated by NDM-1 and CTX-M-15 coproducing *K. pneumoniae* ST11 clone (Baraniak et al., 2016). A high prevalence (30.2%) of CTX-M-15-producing *K. pneumoniae* was detected in raw bovine milk too. This finding highlights the spread of CTX-M-15-producing *K. pneumoniae* also in the food chain (Diab et al., 2017).

In recent years, new drug-resistant international lineages have emerged, among them, KPC-producing *K. pneumoniae* ST307 has been recognized in several countries (Castanheira et al., 2013; Villa et al., 2017). To the best of our knowledge, our study is the first description of ST307 in Hungary that is has been reported as a potential high-risk clone. High similarity has been found in our ST307 isolate compared to that of detected by Villa et al. (2017).

Three pulsotypes were identified among the investigated *K. pneumoniae* strains: KP053, S PT, and KP197. Two ST11 isolates belonged to KP053 (Kpn33 and Kpn125) and the third ST11 was detected as S PT (Kpn115) that was earlier reported in Hungary (Damjanova et al., 2008). In a Hungarian study, PFGE typing revealed 12 pulsotypes; of these, KP053 (262/312) and KP070 (38/312) belonged to sequence type ST11 (Kis et al., 2016); these data also prove the spread of KP053/ST11 clone in our country. *K. pneumoniae* ST307 (Kpn47) was classified as KP197 pulsotype, however, this type was not registered until 2014 by the National Public Health Institute. Since 2015, altogether 30 strains have been identified with this pulsotype in Hungary (unpublished data).

In this study, mutations in gyrase and topoisomerase coding genes and various PMQRs were detected in *K. pneumoniae* and *K. oxytoca*. Of the detected PMQRs in this study *oqxAB* was present in all *K. pneumoniae* clinical isolates but not in *K. oxytoca*. This result can be explained by the fact that the *oqxAB* is a chromosomally-encoded gene in *K. pneumoniae* (Yuan et al., 2012). The *qnrB* genes were observed in *K. pneumoniae* ST11 correlating with the international data (Hidalgo et al., 2013; Jaidane et al., 2018). However, this is the first report of the *qnr* gene in *K. oxytoca* ST52. Regarding plasmid replicon types, the most common replicon was IncFIB, that was present in all ST11, ST52, and ST307, which confirms earlier studies (Anes et al., 2017).

Acquisition of *qnr* determinants can have multiple advantages. In the case of *K. oxytoca*, the presence and expression of *qnrA1* caused reduced susceptibility to quinolones. Levofloxacin and moxifloxacin MIC values of *K. pneumoniae* ST11 and ST307 clones correlated with *qnr* and *oqxAB* expression levels (Figure 3).

Further beneficial effect of Qnr proteins can be explained by the toxin-antitoxin effect. Qnr proteins are considered antitoxins, that protect gyrase and topoisomerase IV enzymes from naturally occuring toxins. This theory was described by Ellington and Woodford (2006) and it can be valid also in internationally disseminated high-risk clones (Ellington and Woodford, 2006). During development of fluoroquinolone resistance PMQR determinants play a role in reduced susceptibility, and they maintain low-level fluoroquinolone resistance (Garoff et al., 2018). Later, by chromosomal mutations in QRDRs high-level fluoroquinolone resistance develops, but PMQR expression is maintained thus, indicating further role of PMQRs such as protection of gyrase and topoisomerase IV enzymes (Tran et al., 2005a,b; Redgrave et al., 2014).

It has been also established that the development of fluoroquinolone resistance is diverse among different clones and in the case of international high-risk *K. pneumoniae* clones the fluoroquinolone resistant strains retain fitness that facilitates their dissemination in hospital environment (Fuzi, 2016). Moreover, Redgrave et al. indicated that fluoroquinolone resistance played a key role in evolutionary success of *K. pneumoniae* clones (Redgrave et al., 2014).

Emergence and possible dissemination of *K. pneumoniae* ST307 in hospital settings raises also public health concerns, therefore continous monitoring of high-risk and potential high-risk clones is necessary.

## Repository Data

Assembled genomes of all investigated strains were deposited in NCBI Genbank under the following accession numbers. Raw sequence data of each strain in this study was submitted to Sequence Read Archive (SRA)

Kpn 33: Bioproject: PRJNA511518, Biosample SAMN10639440
Kox37: Bioproject PRJNA511522, Biosample: SAMN106 39457
Kpn47: Bioproject: PRJNA511523, Biosample: SAMN10639726
Kpn115 Bioproject: PRJNA511524, BioSamples SAMN10639736
Kpn125: Bioproject: PRJNA511525, BioSamples SAMN10639737.

## Author Contributions

JD performed pulsed-field gel electrophoresis, multilocus sequence typing, and handled the manuscript. ID performed pulsed-field gel electrophoresis and whole-genome sequencing. KK identified and handled strains from clinical specimen. BL performed whole-genome sequence analysis. BK performed qPCR, analyzed the data, and handled the manuscript. DS was laboratory chief, contributed to study design, and handled the manuscript.

## Conflict of Interest Statement

The authors declare that the research was conducted in the absence of any commercial or financial relationships that could be construed as a potential conflict of interest.

## Footnotes

1 www.eucast.org

2 http://www.pasteur.fr/mlst/Kpneumoniae.html
